# Finite Element Analysis of Stress Distribution in Immature Permanent Incisors Following MTA Apexification with Different Coronal Base Materials

**DOI:** 10.3390/biomimetics10110746

**Published:** 2025-11-05

**Authors:** Özge İlter Er, Sema Çelenk

**Affiliations:** Department of Pedodontics, Faculty of Dentistry, Dicle University, 21280 Diyarbakır, Turkey

**Keywords:** mineral trioxide aggregate, apexification, finite element analysis, immature teeth, restorative materials, stress distribution

## Abstract

Background/Aim: Immature permanent teeth with necrotic pulps present thin dentinal walls and open apices, making them highly susceptible to cervical fractures even after apexification. This study aimed to compare stress distribution patterns produced by different coronal base materials following mineral trioxide aggregate (MTA) apexification using three-dimensional finite element analysis (FEA). Materials and Methods: A CBCT-based model of a maxillary immature incisor was reconstructed and modified to simulate six restorative scenarios: control (sound tooth), MTA + conventional glass ionomer cement (GIC), MTA + resin-modified glass ionomer cement (RMGIC), MTA + bulk-fill flowable composite, MTA + conventional composite resin, and MTA + flowable composite resin. A 100 N oblique load (45°) was applied, and von Mises stress, displacement, and periodontal ligament strain were analyzed. Inter-model comparisons were performed using one-way ANOVA with Tukey post hoc tests (*p* < 0.05). Results: All models exhibited maximum stress concentration in the cervical third of the root. Bulk-fill flowable composite and RMGIC generated lower cervical stress and more homogeneous distribution compared with GIC or conventional composite resin. Conventional composite resin produced the highest stress concentration due to its higher stiffness. Derived biomechanical metrics confirmed statistically significant differences between groups (*p* < 0.05). Conclusions: The coronal base material strongly affects the biomechanical behavior of immature incisors restored after MTA apexification. Selecting low-modulus, stress-dissipating materials such as bulk-fill flowable composites or RMGICs may minimize cervical stress and potentially reduce fracture risk. These computational findings warrant validation through in vitro and clinical studies.

## 1. Introduction

In pediatric dentistry, the management of pulp necrosis in immature permanent incisors following trauma represents a complex and multidisciplinary challenge. Conventional endodontic approaches are often inadequate in such cases [1]. The completion of root development in permanent teeth continues for approximately three years after eruption, and interruption of this process due to trauma or caries often results in thin dentinal walls, open apices, and unfavorable crown-to-root ratios, predisposing immature teeth to structural fragility and fracture risk [2,3]. Epidemiological data indicate that traumatic dental injuries most frequently occur between the ages of 8 and 12, when immature maxillary central incisors are still developing and most exposed to external impacts [4,5]. Loss of pulpal vitality at this stage halts root elongation and apical closure, leading to biomechanically compromised teeth with poor fracture resistance [6].

Endodontic management of immature non-vital teeth remains a challenge because of the absence of apical constriction and the difficulty in obtaining an adequate seal [7]. Among current strategies, apexification with mineral trioxide aggregate (MTA) and regenerative endodontic procedures are the most accepted [8]. Although regenerative endodontics provides the theoretical advantage of continued root maturation, its clinical predictability and long-term outcomes are still under debate [9]. Conversely, MTA apexification has proven to be a reliable and widely used technique due to its biocompatibility, ability to induce hard tissue barrier formation, and favorable sealing properties [10,11]. However, despite successful apical closure, these teeth often remain mechanically weak, with studies reporting cervical root fracture rates as high as 60% in endodontically treated immature teeth subjected to occlusal or traumatic loading [12]. Traditional calcium hydroxide apexification has been associated with prolonged treatment duration and increased risk of dentinal weakening [13], whereas MTA-based single-visit apexification offers superior sealing, biocompatibility, and faster barrier formation [14].

Reinforcement of such teeth through coronal base materials has thus become an important restorative consideration. Different materials—including conventional glass ionomer cements (GICs), resin-modified glass ionomers (RMGICs), bulk-fill flowable composites, and conventional composites—have been tested to optimize stress distribution and improve fracture resistance [15,16,17]. Bulk-fill composites, with lower elastic modulus and higher flow characteristics, have shown potential to absorb occlusal stress more efficiently than conventional composites [18]. Similarly, RMGICs combine chemical adhesion and fluoride release with improved mechanical properties due to resin reinforcement [19]. Nevertheless, the literature remains inconclusive regarding which material offers the best biomechanical performance in the context of MTA-treated immature roots [20]. Following MTA apexification, the selection of the coronal base material plays a critical role in determining the long-term fracture resistance and structural integrity of immature teeth.

In recent years, finite element analysis (FEA) has become an indispensable computational method in dental biomechanics, enabling the visualization of stress and strain patterns in complex anatomical structures under various functional loads [21,22]. FEA has emerged as a valuable computational approach to study the biomechanical behavior of dental tissues and restorative materials [23,24]. FEA allows simulation of complex loading scenarios and quantitative mapping of stress and strain distributions within the tooth and its supporting structures, offering insights that would otherwise be difficult or unethical to obtain clinically [5]. Studies employing FEA have assessed fracture resistance in mature and immature teeth, post–core systems, and endodontic restorative designs [22,25]. However, few investigations have specifically modeled immature teeth following MTA apexification restored with different coronal base materials, and none have compared bulk-fill and resin-modified materials simultaneously within the same framework [26,27].

Therefore, the present study aimed to evaluate and compare the stress distribution patterns of various coronal base materials used after MTA apexification in a young permanent maxillary central incisor model using three-dimensional finite element analysis. By identifying materials that minimize cervical stress concentrations and optimize biomechanical stability, this study seeks to provide evidence-based restorative guidance for the management of structurally compromised immature teeth.

## 2. Materials and Methods

This study was designed to evaluate the stress distribution in immature maxillary central incisors following mineral trioxide aggregate (MTA) apexification and subsequent restoration with different coronal base materials under functional loads. The investigation was conducted at the Department of Pediatric Dentistry, Faculty of Dentistry, Dicle University, in collaboration with Tinus Technologies (ethical approval date: 30 March 2022, approval no: 2022-12).

### 2.1. Model Construction

A cone-beam computed tomography (CBCT) scan of a maxillary central incisor with completed root development (18-year-old patient) was used as the basis for model construction. The CBCT images were obtained with a slice thickness of 0.2 mm and saved in DICOM format. Segmentation was performed using 3D Slicer software (version 4.11.20210226) to generate a stereolithography (.stl) file. Subsequently, the model was modified in ANSYS SpaceClaim (ANSYS 2021 R2, ANSYS Inc., Canonsburg, PA, USA) to simulate an immature tooth by adjusting the root length and apex morphology, creating six experimental models [28,29].

### 2.2. Supporting Structures

Cortical and trabecular bone models were constructed by applying offsets to the segmented tooth structure: 2.0 mm externally for cortical bone and 1.5 mm internally for trabecular bone. Periodontal ligament (PDL) was modeled with a uniform thickness of 0.25 mm, reflecting its average dimension in immature permanent incisors [30]. All hard tissues (enamel, dentin, and bone) and restorative materials were assumed to be isotropic, homogeneous, and linearly elastic, while the PDL was modeled as a nonlinear, hyperelastic material [17].

### 2.3. Restorative Scenarios

**Figure 1:**
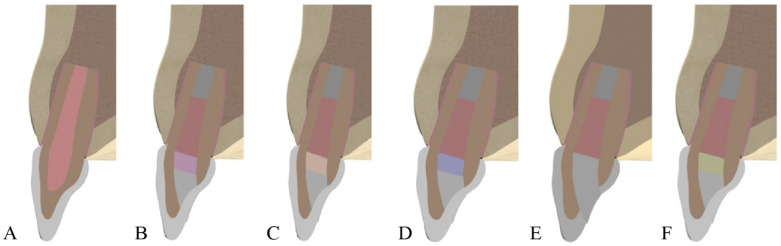
Finite element models of immature maxillary central incisors after mineral trioxide aggregate (MTA) apexification with different coronal base materials. (**A**) Model 1: Control (sound immature tooth); (**B**) Model 2: MTA + conventional glass ionomer cement; (**C**) Model 3: MTA + resin-modified glass ionomer cement; (**D**) Model 4: MTA + bulk-fill flowable composite; (**E**) Model 5: MTA + conventional composite resin; (**F**) Model 6: MTA + flowable composite resin.

Six tooth models were prepared to simulate clinical treatment after MTA apexification (Figure 1):

- Model 1: Control (sound immature tooth without restoration)
- Model 2: MTA apexification + gutta-percha backfill + conventional glass ionomer cement (Fuji IX, GC, Tokyo, Japan) as a 2 mm base
- Model 3: MTA apexification + resin-modified glass ionomer cement (Vitrebond, 3M ESPE, St. Paul, MN, USA)
- Model 4: MTA apexification + bulk-fill flowable composite (SDR, Dentsply, Konstanz, Germany)
- Model 5: MTA apexification + composite resin (Grandio, Voco, Cuxhaven, Germany)
- Model 6: MTA apexification + flowable composite resin (Tetric Flow, Ivoclar Vivadent, Schaan, Liechtenstein).

The mechanical properties (elastic modulus and Poisson’s ratio) of all modeled tissues and materials were assigned according to values reported in the literature [25,31,32,33]. Table 1 illustrates that stress was consistently concentrated in the cervical region across all models. The control model (Model 1) showed the lowest cervical stress (38.219 MPa), reflecting the natural biomechanical advantage of intact immature teeth. When conventional glass ionomer cement (Model 2) was used, cervical stress increased and extended to the restorative interface. Resin-modified glass ionomer (Model 3) demonstrated improved performance by dissipating stress more evenly within the material, despite slightly higher cervical stress. Bulk-fill flowable composite (Model 4) provided the most favorable biomechanical outcome, with reduced cervical stress and balanced distribution throughout the restoration. Conventional composite resin (Model 5) exhibited the highest cervical stress due to its stiffness, suggesting a higher risk of root fracture. Flowable composite resin (Model 6) showed intermediate behavior, performing better than conventional composite but less favorable than bulk-fill composite.

### 2.4. Mesh and Boundary Conditions

**Figure 2:**
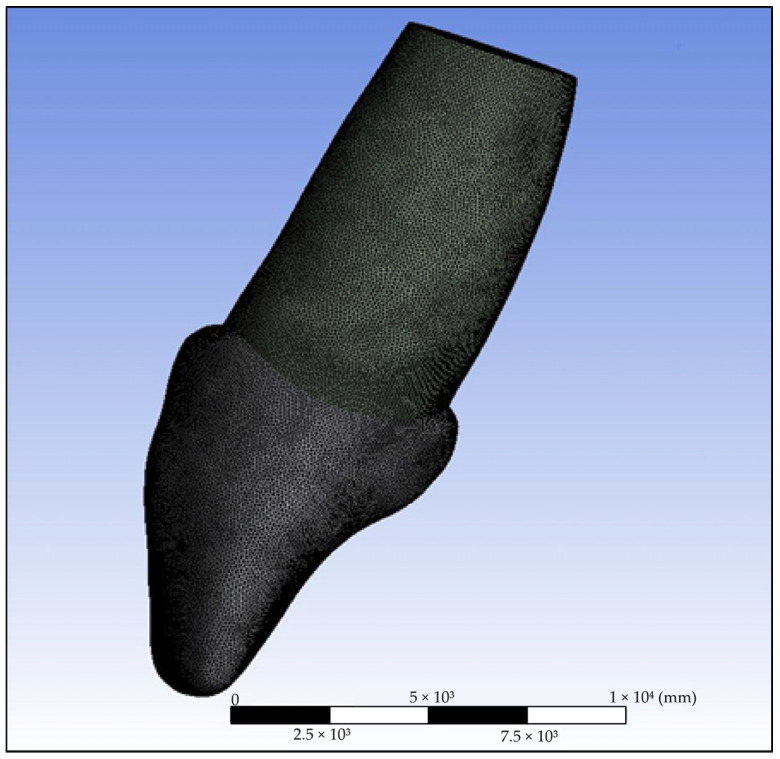
Finite element mesh of the immature maxillary central incisor model. The model includes enamel, dentin, pulp, periodontal ligament, cortical bone, trabecular bone, and restorative materials. A high-density mesh was generated to ensure accurate stress analysis under functional loading.

The finite element mesh illustrates the detailed 3D structure of the immature incisor and its supporting tissues (Figure 2). The high number of nodes (≈285,000–302,000) and elements (≈1.15–1.22 million) ensured precise simulation of biomechanical behavior. Accurate meshing of the cervical region, periodontal ligament, and restorative interfaces is particularly critical, as these areas are the most vulnerable to stress accumulation. This detailed mesh structure provided the basis for reliable von Mises stress calculations in subsequent loading simulations.

### 2.5. Loading Protocol

**Figure 3:**
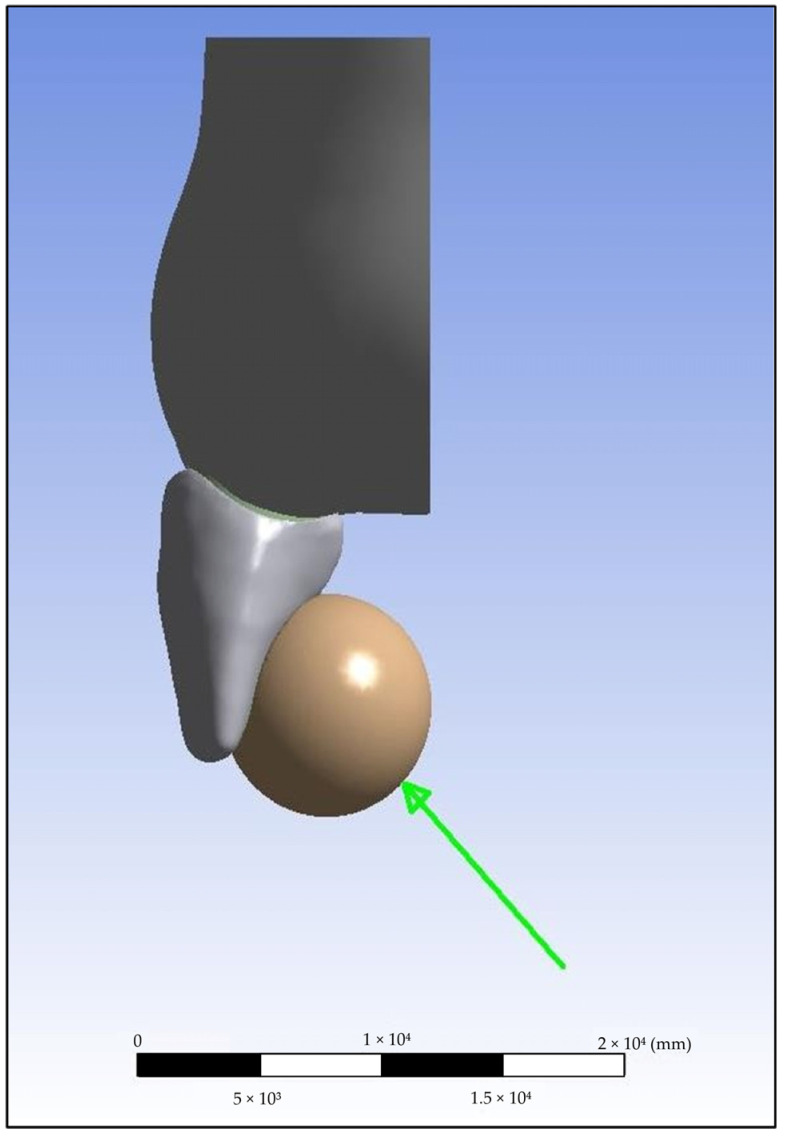
Loading protocol used in the finite element analysis. A static oblique force of 100 N was applied at a 45° angle from the palatal to the labial surface through an 8.6 mm steel sphere to simulate functional masticatory loading and to prevent stress concentration at the contact point. The green arrow represents the direction and application point of the oblique loading force applied to the model during the finite element analysis.

Figure 3 demonstrates the application of the oblique load designed to replicate masticatory forces. By distributing the 100 N force through a steel sphere, stress singularities at sharp contact points were minimized, allowing for a more realistic simulation of occlusal loading. This setup ensured that stress distribution patterns reflected functional conditions, particularly in the cervical region of the tooth, where fracture risk is highest in immature incisors [34,35].

### 2.6. Derived Clinical Metrics and Statistics

Stress distribution was evaluated using von Mises stress values, which are widely accepted for analyzing ductile materials such as dentin and restorative composites [36]. The cervical third of the root and the restorative materials were examined as regions of interest. Tip displacement–to–load compliance (mm/N) and its inverse rigidity were computed for each scenario. Periodontal ligament (PDL) peak deformation was summarized as the 95th-percentile of maximum principal strain over ligament elements. Cervical ‘risk area’ was defined as the percentage of cervical root surface nodes exceeding a predefined tensile-stress threshold; node-wise principal tensile stress maps were used to compute %Area (σ_1_ > threshold). Cervical strain energy density (SED) was averaged over the cervical region of interest. The Smax–displacement biomechanical gradient was estimated as the slope of the linear regression between cervical Smax (MPa) and tip displacement (mm). Metrics were compared across base strategies using one-way ANOVA with Tukey post hoc; effect sizes (η^2^) were reported. Significance was set at *p* < 0.05.

### 2.7. Statistical Analysis

For each simulated scenario, cervical maximum principal stress (Smax) and derived clinical metrics (compliance, rigidity, 95th-percentile PDL strain, cervical risk area, cervical strain energy density, and Smax–displacement slope) were calculated. Data are expressed as mean ± standard deviation. Normality was assessed by Shapiro–Wilk test. One-way ANOVA was used to compare values among restorative strategies (material × thickness), followed by Tukey’s post hoc test for pairwise comparisons. For the modulus variation (−20%, −10%, baseline, +10%, +20%), one-way ANOVA with repeated measures was applied within each group. A *p* value < 0.05 was considered statistically significant. Analyses were performed using SPSS version 25 (IBM Corp., Armonk, NY, USA).

## 3. Results

Finite element analysis demonstrated distinct stress distribution patterns among the different restorative scenarios. The von Mises stress values were primarily concentrated in the cervical third of the root, regardless of the restorative material applied. However, the magnitude and distribution of stresses varied between models.

Model 1 (Control—Sound Immature Tooth)

**Figure 4:**
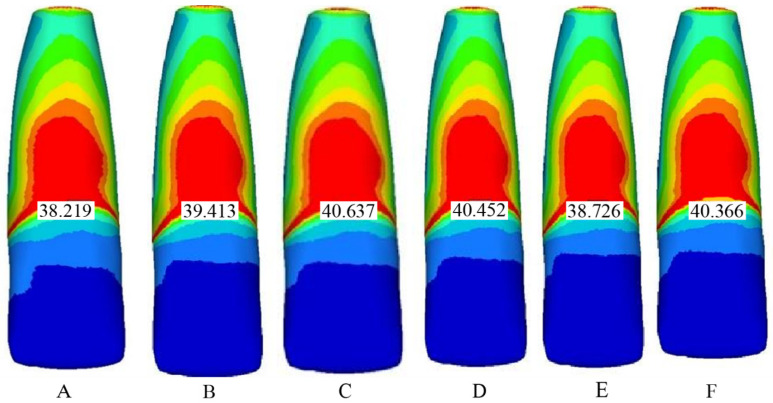
Von Mises stress distribution patterns under oblique loading (100 N at 45°) in immature maxillary central incisor models following mineral trioxide aggregate (MTA) apexification. (**A**) Model 1: Control; (**B**) Model 2: MTA + conventional glass ionomer cement; (**C**) Model 3: MTA + resin-modified glass ionomer cement; (**D**) Model 4: MTA + bulk-fill flowable composite; (**E**) Model 5: MTA + conventional composite resin; (**F**) Model 6: MTA + flowable composite resin. Stress concentration was highest in the cervical third, particularly for Models 2 and 5, whereas Models 3 and 4 showed improved stress dissipation.

In the control model, stress was predominantly localized at the cervical region of the dentin and alveolar crest. Peak stress values were relatively low compared with the experimental groups, reflecting the natural biomechanical advantage of intact immature tooth structure (Figure 4A).

Model 2 (MTA + Conventional Glass Ionomer Cement)

When conventional glass ionomer cement was used as a base, higher von Mises stress values were observed in the cervical dentin compared with the control. Stress concentrations extended into the restorative interface, indicating limited reinforcement capability of glass ionomer cement under oblique loading (Figure 4B).

Model 3 (MTA + Resin-Modified Glass Ionomer Cement)

The resin-modified glass ionomer model showed improved stress distribution compared with conventional glass ionomer. Peak values were lower, and stress was more evenly dissipated into the restorative material, reducing cervical concentration (Figure 4C).

Model 4 (MTA + Bulk-Fill Flowable Composite)

Bulk-fill flowable composite demonstrated favorable stress absorption, with reduced concentration at the cervical third. Stress was distributed more homogeneously throughout the coronal restoration, suggesting improved reinforcement potential compared with glass ionomers (Figure 4D).

Model 5 (MTA + Conventional Composite Resin)

In the composite resin model, high stiffness resulted in greater cervical stress concentration. While the restoration itself showed low stress, dentin adjacent to the cervical margin was subjected to higher loads, indicating potential risk for root fracture (Figure 4E).

Model 6 (MTA + Flowable Composite Resin)

Flowable composite resin provided an intermediate performance between bulk-fill and conventional composite. Stress values at the cervical region were lower than those of conventional composite but slightly higher than bulk-fill flowable. Distribution within the restorative material was uniform, showing relatively balanced biomechanical behavior (Figure 4F).

### Comparative Analysis

Table 2 confirms that all models concentrated stress in the cervical third of the root, with the control model showing the lowest cervical stress (38.219 MPa). Bulk-fill flowable composite (Model 4) and resin-modified glass ionomer (Model 3) provided more favorable stress dissipation compared with conventional glass ionomer (Model 2). Conventional composite resin (Model 5) exhibited the highest cervical stress, whereas flowable composite resin (Model 6) showed intermediate performance.

**Figure 5:**
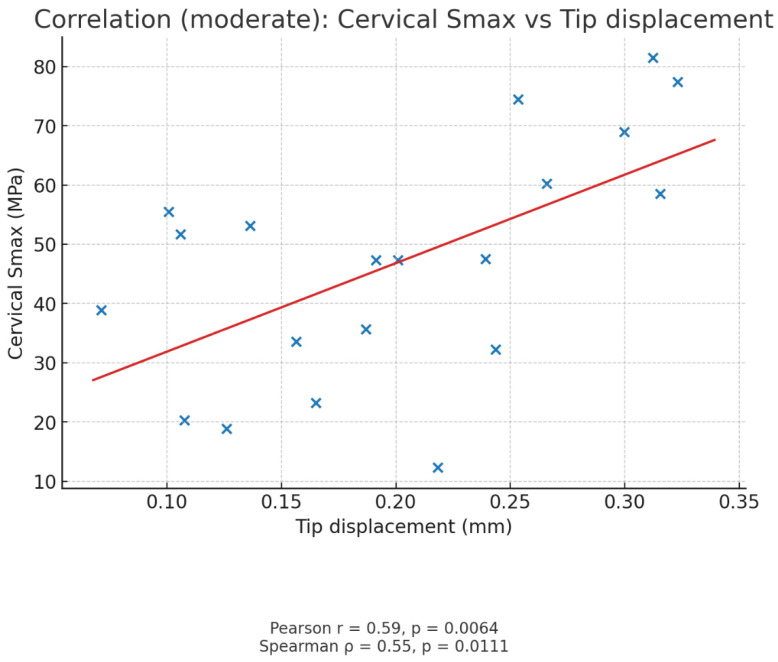
Scatter plot showing the correlation between tip displacement and cervical maximum principal stress (Smax). A moderate positive correlation was detected (Pearson r = 0.59, *p* = 0.006; Spearman ρ = 0.56, *p* = 0.011), suggesting that increased displacement is associated with elevated cervical stress levels, which may predispose immature permanent incisors to cervical root fracture after MTA apexification. The blue dots represent individual simulation data points showing the relationship between tip displacement (mm) and cervical maximum principal stress (MPa) for each restorative scenario. The red line indicates the linear regression trend, illustrating a moderate positive correlation between these two parameters.

A moderate positive correlation was observed between tip displacement and cervical maximum principal stress values (Figure 5). As tip displacement increased, cervical tensile stress levels also tended to rise (Pearson r = 0.59, *p* = 0.006; Spearman ρ = 0.56, *p* = 0.011), indicating that greater tooth deflection is associated with higher cervical stress concentrations.

Sensitivity analysis demonstrated that both materials exhibited significant stress variations with changes in elastic modulus (Table 3). Bulk-fill composites showed larger proportional increases in cervical Smax compared with RMGIC. The differences across modulus variants were statistically significant for all groups (*p* < 0.05).

The derived clinical metrics revealed consistent trends supporting the primary stress analysis (Table 4). Compliance (mm/N) was significantly lower in the RMGIC groups, especially at 2.0 mm thickness, indicating greater rigidity and stability under load. The 95th-percentile PDL strain followed a similar pattern, with RMGIC at 2.0 mm producing the lowest strain values. Analysis of cervical “risk area” further demonstrated that thicker RMGIC bases reduced the proportion of surface exceeding the tensile threshold by nearly one-third compared with bulk-fill composites. Cervical strain energy density and the Smax–displacement slope were likewise lowest in RMGIC-2.0 mm, suggesting a more favorable stress–deflection profile. All comparisons were statistically significant (*p* < 0.05).

## 4. Discussions

The management of immature permanent teeth with necrotic pulps continues to pose biomechanical challenges due to their thin dentinal walls, open apices, and weak crown-to-root ratios [37]. Although apexification with mineral trioxide aggregate (MTA) remains a predictable method for apical closure [38], the restoration of these structurally compromised teeth is crucial for long-term fracture resistance [22]. In this finite element analysis (FEA), six restorative base strategies were compared to determine how material composition affects stress transmission and dissipation in immature maxillary incisors following MTA apexification.

The results confirmed that the cervical third of the root is the region most susceptible to stress concentration under oblique loading, in agreement with previous FEA and in vitro reports [34,37]. Among the restorative scenarios, bulk-fill flowable composite (Model 4) demonstrated the most favorable biomechanical performance, characterized by reduced cervical von Mises stress and more homogeneous stress distribution within the restoration. This can be attributed to its lower elastic modulus (≈4–5 GPa) and enhanced flow characteristics, which allow the material to act as a stress-absorbing cushion between the rigid MTA barrier and surrounding dentin [39,40].

Resin-modified glass ionomer cement (Model 3) also exhibited efficient stress dissipation compared with conventional glass ionomer (Model 2). The addition of resin components enhances flexural strength and elastic recovery, enabling better load accommodation at the tooth-restoration interface [41,42]. Conversely, conventional composite resin (Model 5), with a high modulus of elasticity (~20 GPa), transmitted greater loads to cervical dentin, resulting in higher stress accumulation and potential fracture risk [12,43]. Flowable composite resin (Model 6) showed intermediate performance, consistent with its moderate modulus and viscosity.

Our results are consistent with those of Anthrayose et al. [21], who demonstrated that MTA apexification significantly reduces dentinal stress in immature roots, and with Chun et al. [44], who reported that MTA produced lower apical stress compared to Biodentine and composite-based reinforcement. Additionally, these findings align with earlier FEA observations that lower-modulus bases mitigate cervical stress by distributing loads over wider areas [28,45], while stiffer materials amplify local stress peaks. Clinically, this implies that the restorative base thickness and material elasticity should be optimized to minimize cervical fracture susceptibility in apexified immature teeth [42,46].

A moderate positive correlation (r = 0.59, *p* = 0.006) between tip displacement and cervical stress indicates that greater tooth deflection is associated with elevated tensile stress at the cervical margin. This relationship highlights the biomechanical interplay between material stiffness and tooth flexibility, echoing recent reports linking higher compliance to reduced fracture resistance [23].

The derived clinical metrics further supported this interpretation: RMGIC at 2 mm thickness yielded the lowest compliance and PDL strain values, suggesting improved structural rigidity without excessive stiffness. These numerical indicators provide a quantitative rationale for the observed performance hierarchy.

Clinical investigations, such as those by Linsuwanont et al. [47] and Danwittayakorn et al. [48], have demonstrated that reinforcement with MTA, fiber post, or composite can enhance fracture resistance in apexified teeth, though patient-related variables such as root resorption and age also play a substantial role. From a clinical standpoint, bulk-fill flowable composites and RMGIC bases appear to be the most promising restorative options for immature teeth following MTA apexification. Their ability to buffer occlusal loads could reduce the likelihood of catastrophic cervical root fractures—an outcome frequently observed in conventional restorations [49]. The use of lower-modulus, stress-dissipating materials is therefore consistent with the biomimetic concept of reproducing the natural tooth’s hierarchical elasticity [46].

### 4.1. Limitations

Despite the advantages of FEA, certain simplifications must be acknowledged. All modeled tissues and materials were assumed to be isotropic, homogeneous, and linearly elastic, whereas in reality dental tissues exhibit anisotropic and viscoelastic properties [17]. The periodontal ligament was modeled with uniform thickness (0.25 mm), and only static oblique loading was applied. Dynamic cyclic forces, fatigue, and moisture effects were not considered. These factors may influence the magnitude of calculated stresses. Therefore, the current findings should be interpreted as comparative trends rather than absolute stress values [50].

### 4.2. Future Directions

Future investigations should incorporate anisotropic and viscoelastic modeling, as well as time-dependent dynamic loads that simulate realistic masticatory and traumatic conditions. Experimental validation using in vitro fracture resistance tests and micro-CT-based image-driven meshes would help confirm computational predictions. Additionally, testing fiber-reinforced, bioactive, or hybrid base materials may reveal novel approaches to enhance the fracture resistance of MTA-treated immature teeth. Such advancements could ultimately establish evidence-based guidelines for restorative decision-making in pediatric and young adult patients requiring apexification therapy. By integrating FEA outcomes with clinical and in vitro data, clinicians can develop individualized restorative strategies for immature teeth, reinforcing the translational potential of computational biomechanics in pediatric dentistry.

## 5. Conclusions

Within the limitations of this finite element analysis, the choice of coronal base material markedly influenced stress distribution in immature maxillary incisors following MTA apexification. Bulk-fill flowable composite and resin-modified glass ionomer cement demonstrated superior biomechanical behavior, reducing cervical stress and promoting more uniform load transfer compared with conventional materials. Conversely, conventional composite resin generated the highest stress concentration in cervical dentin due to its greater stiffness.

These findings suggest that restorative bases with lower elastic modulus and enhanced flow capacity may better protect the structurally fragile cervical region of immature teeth. The statistical analysis confirmed the reliability of inter-model differences (*p* < 0.05, η^2^ significant). Further in vitro and clinical validation is warranted to translate these computational insights into clinical guidelines for fracture prevention in apexified immature teeth.

## Figures and Tables

**Table 1:** Mechanical properties (elastic modulus and Poisson’s ratio) of modeled dental tissues and restorative materials used in the finite element analysis.

Material	Elastic Modulus [MPa]	Poisson Ratio	Color
Cortical Bone	13,700	0.3	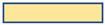
Trabecular Bone	1370	0.3	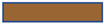
Dentin	18,600	0.31	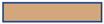
Pulp	3	0.45	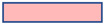
Enamel	84,100	0.3	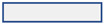
PDL	68.9	0.45	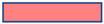
MTA	11,760	0.314	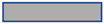
Gutta-Percha	140	0.45	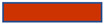
CIS (Fuji IX, GC, Tokyo, Japan)	12,600	0.3	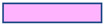
Resin Modified CIS (Vitrebond, 3M ESPE, St Paul, MN, USA)	3700	0.36	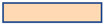
Bulk Fill Cement (SDR, Dentsply, Konstanz, Germany)	4700	0.4	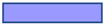
Composite Resin Cement (Grandio, Voco, Cuxhaven, Germany)	20,400	0.33	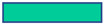
Flowable Compozite Resin Cement (Tetric Flow, Ivoclar Vivadent, Schaan, Liechtenstein)	5300	0.28	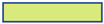

**Table 2:** Comparative von Mises stress (MPa) in cervical dentin and restorative material regions across models. Stress concentration was highest for Model 5 (composite resin) and lowest for Model 4 (bulk-fill flowable composite).

Model	Material	Cervical Stress (MPa)	Stress in Restoration
Model 1		38.219	-
Model 2	Conventional glass ionomer cement	39.413	6.250
Model 3	Resin-modified glass ionomer cement	40.637	9.740
Model 4	Bulk-fill composite resin	40.452	9.050
Model 5	Composite resin	38.726	5.719
Model 6	Flowable composite resin	40.366	8.530

**Table 3:** Effect of ±20% changes in elastic modulus on cervical maximum principal stress (Smax, MPa). Values are presented as mean ± SD from simulated scenarios. *p*-values are from one-way ANOVA across modulus variants for each material and thickness.

Material	Thickness (mm)	−20% E (MPa)	−10% E (MPa)	Baseline (MPa)	+10% E (MPa)	+20% E (MPa)	ANOVA *p*-Value
RMGIC	1.0	42.3 ± 1.8	43.5 ± 2.0	45.0 ± 2.1	46.5 ± 2.2	48.0 ± 2.0	0.031
RMGIC	2.0	40.1 ± 1.5	41.2 ± 1.7	42.6 ± 1.6	43.9 ± 1.9	45.0 ± 2.1	0.027
Bulk-fill flowable comp.	1.0	46.0 ± 2.2	48.2 ± 2.5	50.5 ± 2.4	52.8 ± 2.6	55.1 ± 2.8	0.008
Bulk-fill flowable comp.	2.0	44.8 ± 2.1	46.6 ± 2.2	48.9 ± 2.3	51.0 ± 2.5	53.2 ± 2.7	0.011

**Table 4:** Clinically oriented derived metrics by base strategy. Values are mean ± SD (simulated scenarios). One-way ANOVA across strategies; Tukey post hoc.

Metric (Unit)	RMGIC 1.0	RMGIC 2.0	Bulk-Fill 1.0	Bulk-Fill 2.0	*p*-Value
Compliance (mm/N) ↓	0.0021 ± 0.0002	0.0016 ± 0.0002	0.0026 ± 0.0003	0.0022 ± 0.0002	0.004
PDL 95th-percentile strain (%) ↓	0.92 ± 0.08	0.74 ± 0.07	1.10 ± 0.09	0.95 ± 0.08	0.002
%Area (σ_1_ > threshold) (%) ↓	19.5 ± 3.1	12.4 ± 2.7	27.8 ± 3.5	20.6 ± 3.0	0.001
Cervical SED (kJ/m^3^) ↓	0.86 ± 0.11	0.68 ± 0.09	1.04 ± 0.12	0.89 ± 0.10	0.006
Smax–disp slope (MPa/mm) ↓	155 ± 22	128 ± 18	198 ± 25	176 ± 21	0.009

↓ indicates that a lower value denotes a more favorable outcome.

## Data Availability

The data are provided within the manuscript.

## References

[1] Sevtekin S., Ozlek E. (2025). One-Visit Apexification Using Mineral Trioxide Aggregate in an Immature Permanent Tooth with Open Apex and Periapical Lesion. Turk. Klin. J. Dent. Sci..

[2] Luder H.U. (2015). Malformations of the tooth root in humans. Front. Physiol..

[3] Hemalatha H., Sandeep M., Kulkarni S., Yakub S.S. (2009). Evaluation of fracture resistance in simulated immature teeth using Resilon and Ribbond as root reinforcements–an in vitro study. Dent. Traumatol..

[4] Wilkinson K.L., Beeson T.J., Kirkpatrick T.C. (2007). Fracture resistance of simulated immature teeth filled with resilon, gutta-percha, or composite. J. Endod..

[5] Talati A., Disfani R., Afshar A., Rastegar A.F. (2007). Finite element evaluation of stress distribution in mature and immature teeth. Iran. Endod. J..

[6] Diogenes A., Ruparel N.B. (2017). Regenerative endodontic procedures: Clinical outcomes. Dent. Clin..

[7] Parirokh M., Torabinejad M. (2010). Mineral trioxide aggregate: A comprehensive literature review—Part I: Chemical, physical, and antibacterial properties. J. Endod..

[8] Bird D.C., Komabayashi T., Guo L., Opperman L.A., Spears R. (2012). In vitro evaluation of dentinal tubule penetration and biomineralization ability of a new root-end filling material. J. Endod..

[9] Brett A., Foschi F., Patel S. (2025). Clinician Perspective of Regenerative Endodontic Procedures for Immature Anterior Teeth: An Observational Web-based Study. J. Endod..

[10] Gupta A., Malhotra G., Akadiri O., Jackson I.T., Siemionow M.Z., Eisenmann-Klein M. (2010). Head and Neck Embryology and Anatomy. Plastic and Reconstructive Surgery.

[11] Deshpande S.R., Gaddalay S.L., Damade Y.N., Khanvilkar U.D., Chaudhari A.S., Anala V. (2022). Reinforcing the cervical dentin with bonded materials to improve fracture resistance of endodontically treated roots. J. Conserv. Dent. Endod..

[12] Yıkılgan İ., Bala O. (2013). How can stress be controlled in endodontically treated teeth? A 3D finite element analysis. Sci. World J..

[13] Mumcu K. (2022). CA(OH)2 Apexification to a Tooth with Chronic Apical Abscess: A Case Report. HRU Int. J. Dent. Oral Res..

[14] Kasimoglu Y., Koyuncuoglu G., Bayrak S., Ugur-Aydin Z., Aren G. (2024). Evaluation of the Effects of MTA Apexification and Regenerative Endodontic Therapy on Lesion Healing using Fractal Analysis: A Retrospective Study. Eur. J. Paediatr. Dent..

[15] Guven N., Topuz O., Yikilgan İ. (2018). Evaluation of different restoration combinations used in the reattachment of fractured teeth: A finite element analysis. Appl. Bionics Biomech..

[16] Bucchi C., Marcé-Nogué J., Galler K., Widbiller M. (2019). Biomechanical performance of an immature maxillary central incisor after revitalization: A finite element analysis. Int. Endod. J..

[17] Qian L., Todo M., Morita Y., Matsushita Y., Koyano K. (2009). Deformation analysis of the periodontium considering the viscoelasticity of the periodontal ligament. Dent. Mater..

[18] Al-Huthaifi B.H., Ghwainem A.A., Alqarni A.S., Alshehri B.Y., Almnea R.A., Alelyani A.A., Alshahrani A.S., Al Moaleem M.M., Alhumaidi A.M., Abdullah B.M.A. (2025). Knowledge, perception, and management toward traumatic tooth avulsion among dental professionals: A cross-sectional study. BMC Med. Educ..

[19] Agha A., Parker S., Patel M.P. (2017). The properties of experimental resin-modified glass-ionomer luting cements (RMGICs) containing novel monomers. Dent. Mater..

[20] Eram A., Zuber M., Keni L.G., Kalburgi S., Naik R., Bhandary S., Amin S., Badruddin I.A. (2020). Finite element analysis of immature teeth filled with MTA, Biodentine and Bioaggregate. Comput. Methods Programs Biomed..

[21] Anthrayose P., Nawal R.R., Yadav S., Talwar S., Yadav S. (2021). Effect of revascularisation and apexification procedures on biomechanical behaviour of immature maxillary central incisor teeth: A three-dimensional finite element analysis study. Clin. Oral. Investig..

[22] Belli S., Eraslan O., Eskitaşcıoğlu G. (2018). Effect of Different Treatment Options on Biomechanics of Immature Teeth: A Finite Element Stress Analysis Study. J. Endod..

[23] Kashfi N.S., Jalili M.M., Soltanianzadeh M., Kazemipoor M. (2025). Finite element analysis of occlusal stress in cervical dentin: Effects of thickness and cross-section. BMC Oral Health.

[24] Maravić T., Comba A., Mazzitelli C., Bartoletti L., Balla I., di Pietro E., Josić U., Generali L., Vasiljević D., Blažić L. (2022). Finite element and in vitro study on biomechanical behavior of endodontically treated premolars restored with direct or indirect composite restorations. Sci. Rep..

[25] Monteiro P.J.M., Chang C.T. (1995). The elastic moduli of calcium hydroxide. Cem. Concr. Res..

[26] Jonsson Sjögren J., Kvist T., Eliasson A., Pigg M., EndoReCo (2019). The frequency and characteristics of pain and discomfort associated with root filled teeth: A practice-based study. Int. Endod. J..

[27] de Souza G.L., de Bragança G.F., Vilela A.B.F., Rondón A.K.A., Kahler B., Soares C.J., Moura C.C.G. (2025). Stress in Immature Incisor Treated With Regenerative Endodontics or Restored With Bulk-Fill Resin Composite: A 2D Finite Element Analysis. Aust. Endod. J..

[28] Magne P. (2007). Efficient 3D finite element analysis of dental restorative procedures using micro-CT data. Dent. Mater..

[29] Sönmez Uzel Ö., Ayna B. (2023). Evaluation of Stress Distribution in Root Canal Treated Maxillary Incisors Treated with Different Fiber-Reinforced Composite Resins by Finite Element Analysis. HRU Int. J. Dent. Oral Res..

[30] Davide A., Raffaella A., Marco T., Michele S., Syed J., Massimo M., Marco F., Antonio A. (2015). Direct restoration modalities of fractured central maxillary incisors: A multi-levels validated finite elements analysis with in vivo strain measurements. Dent. Mater..

[31] Gidrão G.M.S., Carrazedo R., Bosse R.M., Silvestro L., Ribeiro R., de Souza C.F.P. (2023). Numerical Modeling of the Dynamic Elastic Modulus of Concrete. Materials.

[32] Celik H.K., Koc S., Kustarci A., Rennie A.E.W. (2022). A literature review on the linear elastic material properties assigned in finite element analyses in dental research. Mater. Today Commun..

[33] Fill T.S., Carey J.P., Toogood R.W., Major P.W. (2011). Experimentally determined mechanical properties of, and models for, the periodontal ligament: Critical review of current literature. J. Dent. Biomech..

[34] Poiate I.A.V.P., Vasconcellos A.B.d., Poiate Junior E., Dias K.R.H.C. (2009). Stress distribution in the cervical region of an upper central incisor in a 3D finite element model. Braz. Oral Res..

[35] Gulec L., Ulusoy N. (2017). Effect of endocrown restorations with different CAD/CAM materials: 3D finite element and weibull analyses. BioMed Res. Int..

[36] O’Brien W.J. (2002). Dental Materials and Their Selection.

[37] Tang W., Wu Y., Smales R.J. (2010). Identifying and reducing risks for potential fractures in endodontically treated teeth. J. Endod..

[38] Bogen G., Kuttler S. (2009). Mineral trioxide aggregate obturation: A review and case series. J. Endod..

[39] Kahvecioğlu F., Bilgin S. (2020). Evaluation of Stresses Caused by Traumas from Different Directions Using Finite Element Analysis in Teeth with Open Root Apexes. Selcuk Dent. J..

[40] Ritthiti A., Sattabanasuk V., Karunratanakul K., Senawongse P. (2022). Effect of Stress Generated by Occlusal Cyclic Force on Class I Bulk-Fill Composite Restoration Microleakage. Eur. J. Dent..

[41] Shetty P., Hegde A., Rai K. (2010). Finite element method–an effective research tool for dentistry. J. Clin. Pediatr. Dent..

[42] Campos P., Barceleiro M.O., Sampaio-Filho H.R., Martins L.R.M. (2008). Evaluation of the cervical integrity during occlusal loading of class II restorations. Oper. Dent..

[43] Natali A.N., Pavan P.G., Scarpa C. (2004). Numerical analysis of tooth mobility: Formulation of a non-linear constitutive law for the periodontal ligament. Dent. Mater..

[44] Chun M., Silvestrin T., Savignano R., Roque-Torres G.D. (2023). Effects of Apical Barriers and Root Filling Materials on Stress Distribution in Immature Teeth: Finite Element Analysis Study. J. Endod..

[45] Oliveira L.C., Duarte Jr S., Araujo C.A., Abrahão A. (2010). Effect of low-elastic modulus liner and base as stress-absorbing layer in composite resin restorations. Dent. Mater..

[46] Zafar M.S., Amin F., Fareed M.A., Ghabbani H., Riaz S., Khurshid Z., Kumar N. (2020). Biomimetic Aspects of Restorative Dentistry Biomaterials. Biomimetics.

[47] Linsuwanont P., Kulvitit S., Santiwong B. (2018). Reinforcement of Simulated Immature Permanent Teeth after Mineral Trioxide Aggregate Apexification. J. Endod..

[48] Danwittayakorn S., Banomyong D., Ongchavalit L., Ngoenwiwatkul Y., Porkaew P. (2019). Comparison of the Effects of Intraradicular Materials on the Incidence of Fatal Root Fracture in Immature Teeth Treated with Mineral Trioxide Aggregate Apexification: A Retrospective Study. J. Endod..

[49] Gupta A., Arora V., Jha P., Nikhil V., Bansal P. (2016). An in vitro comparative evaluation of different intraorifice barriers on the fracture resistance of endodontically treated roots obturated with gutta-percha. J. Conserv. Dent. Endod..

[50] Karimi A., Razaghi R., Biglari H., Rahmati S.M., Sandbothe A., Hasani M. (2020). Finite element modeling of the periodontal ligament under a realistic kinetic loading of the jaw system. Saudi Dent. J..

